# Improved Catalyst Performance for the Oxygen Evolution Reaction under a Chiral Bias

**DOI:** 10.1021/acscatal.4c04477

**Published:** 2024-11-11

**Authors:** Aravind Vadakkayil, Wiley A. Dunlap-Shohl, Meera Joy, Brian P. Bloom, David H. Waldeck

**Affiliations:** Chemistry Department, University of Pittsburgh, Pittsburgh, Pennsylvania 15260, United States

**Keywords:** oxygen evolution reaction, chiral induced spin selectivity, water splitting, spin catalysis, Monte Carlo model

## Abstract

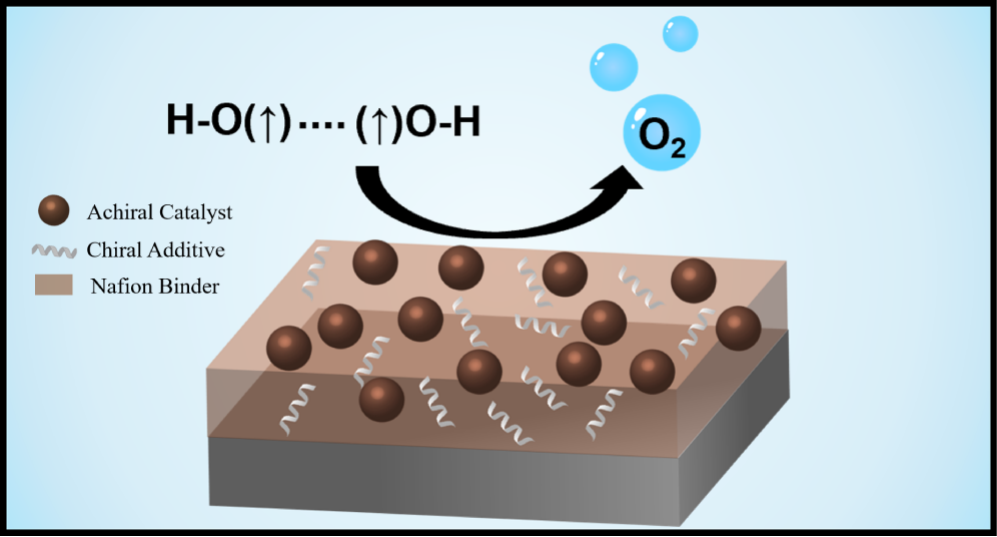

The oxygen evolution reaction (OER) remains an important bottleneck for widespread implementation of a hydrogen economy. While improvements in the OER can be realized by spin polarizing the reaction intermediates, these methods often rely on applying external magnetic fields to ferromagnetic catalysts or by adsorbing chiral molecules onto the catalyst. Here, we show that the addition of chiral additives to the conductive binder supporting the catalysts enhances the selectivity for O_2_ formation and results in exceedingly high mass activities. The results are explained within the context of a statistical model in which the additives are hypothesized to act as a localized chiral bias that enhances radical intermediate coupling. More broadly, these studies illustrate a flexible design motif for improving OER catalysis that persists under different pH conditions, is independent of the choice of catalyst, and can be extrapolated to other chemical reactions.

## Introduction

Recent studies suggest that control over the electron spin degree of freedom can be leveraged to direct chemical reactions and to affect stereochemistry.^1−4^ For the oxygen evolution reaction (OER), reports show that spin-polarized surfaces can lower reaction overpotentials, even shifting the rate-determining step,^5,6^ and improve the Faradaic efficiency,^7^ i.e., the fraction of current attributable to the desired reaction pathway. The OER is believed to proceed through a complex series of four proton-coupled electron transfer steps with adsorbed *OH, *O, and *OOH radical intermediates and ending with diatomic oxygen, which in its electronic ground state exists as a triplet.^8,9^ Theoretical models suggest that spin polarization during catalysis can modify transition state energies for reaction intermediates, as well as the reaction coordinate, i.e., identity and configuration of adsorbates, compared to systems with unpolarized catalysts.^5,10,11^

Two primary modalities have been employed to achieve spin polarization during OER electrocatalysis. In one approach, the application of an external magnetic field on a paramagnetic or ferromagnetic catalyst is used to improve OER performance.^5,10,12−14^ This approach is limited to catalyst materials that are ferromagnetic or paramagnetic, however, unless “spin pinning” layers are also incorporated,^10,15^ and requires a permanent applied magnetic field. The second major approach is to use chiral catalysts that display the chiral induced spin selectivity (CISS) effect.^16−21^ The CISS effect refers to the intrinsic attribute of chiral materials to act as “spin filters” during current transport,^22−24^ yet only a few such materials have been shown to be suitable for OER. Other, less common, approaches exploit spin-selective carrier properties, either inherent to the catalyst or through engineering spin polarized defect sites on the catalyst.^25−27^ These constraints hamper the widespread employment of spin polarization for catalysis studies.

This work demonstrates an alternative approach, in which molecular additives are used to create a chiral bias that promotes formation of spin-polarized intermediates. The chiral molecular additives are combined with traditional catalyst binder supports and are not redox active at OER potentials. This approach sidesteps the need to reengineer a chiral catalyst from its achiral analogue. The versatility of the design motif is demonstrated for three different catalysts and the improvements in OER efficiency are found to be on par with or larger than that of traditional strategies for creating spin polarization using chiral catalysts. Moreover, the favorable characteristics are shown to persist over a wide range of solution pH conditions.

## Results and Discussion

**Scheme 1:**
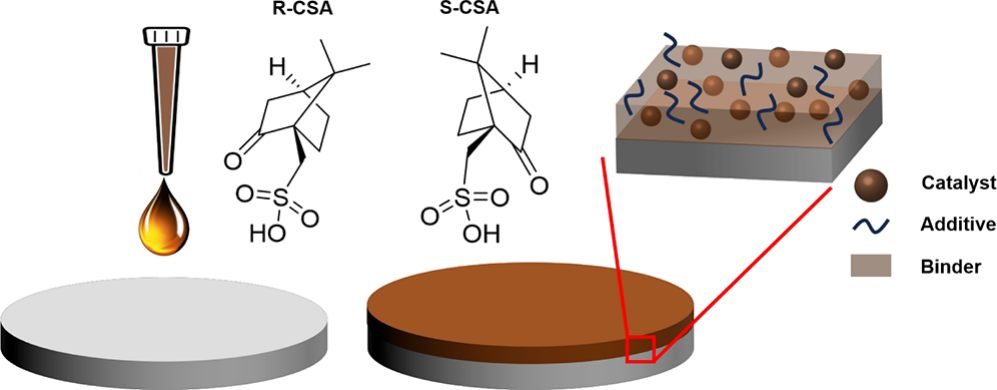
Schematic Illustrates the Process for the Formation of Thin Film Electrocatalysts An ink suspension comprising nanoparticle catalysts, an additive, and a Nafion binder are drop-cast onto a glassy carbon electrode surface to create a thin film. Subsequently, the electrode film is heated in an oven to remove the solvent. See the Supporting Information for more details.

Scheme 1 illustrates the architecture and preparation method for the electrocatalyst films. Here, ink solutions comprising a molecular additive, an achiral catalyst, and a binder support are used to prepare catalyst films on an electrode surface. The effect of chirality on the electrocatalytic performance was evaluated by comparing films that were prepared from inks with chiral molecular additives to those with no molecular additives and with racemic mixtures of molecular additives. See Methods section for additional details regarding preparation and Table S3 for molecular structures.

**Figure 1:**
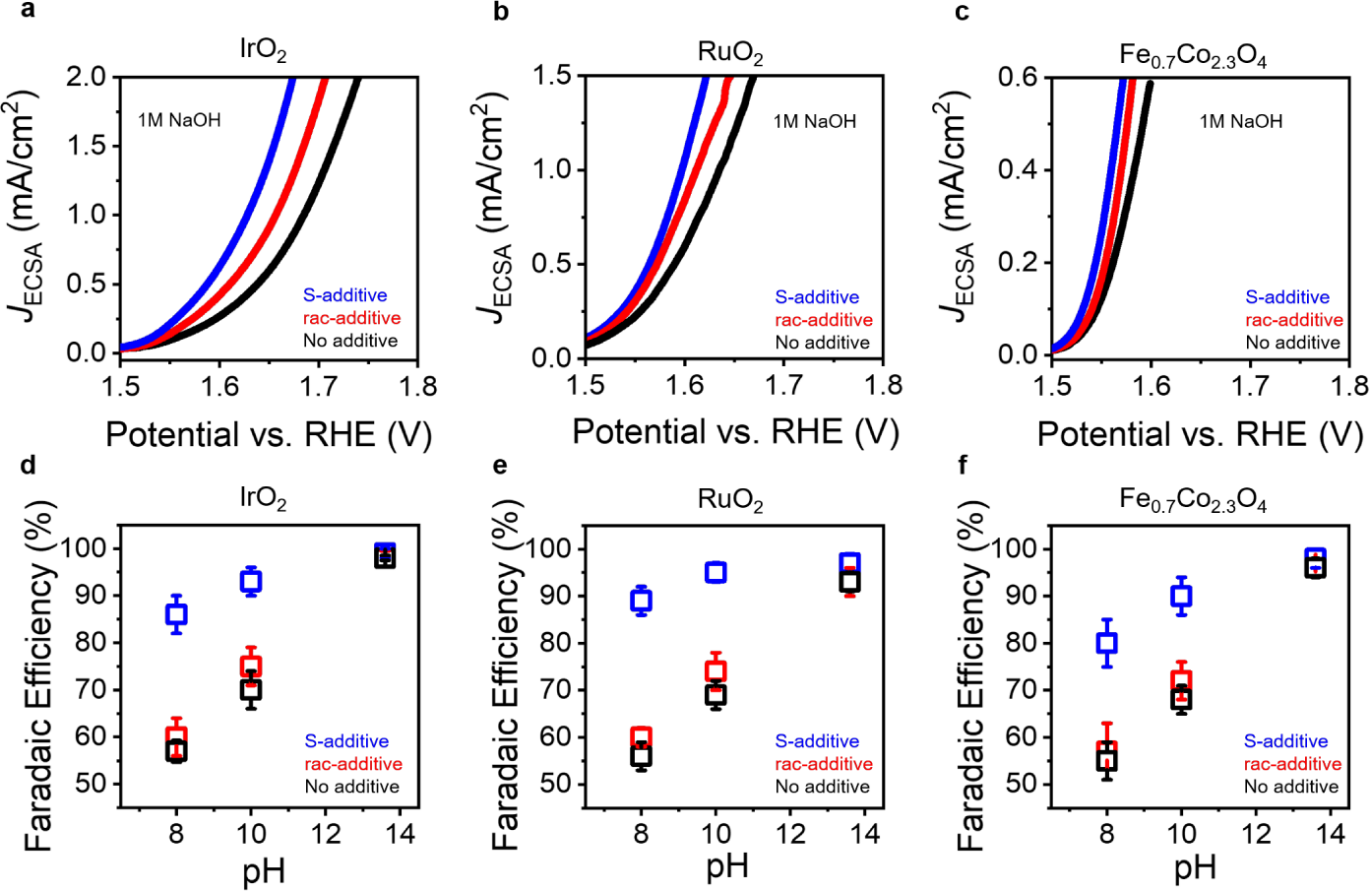
Effect of chiral additives on the oxygen evolution reaction. Linear sweep voltammograms of IrO_2_ (a), RuO_2_ (b), and Fe_0.7_Co_2.3_O_4_ (c) catalysts prepared with S-CSA additives (blue), rac-CSA (red) additive mixtures, and in the absence of additives to the Nafion binder (black). The curves in panels a–c represent the average of three independently prepared electrodes. Panels d–f plot the Faradaic efficiency of IrO_2_, RuO_2_, and Fe_0.7_Co_2.3_O_4_ catalysts prepared with S-CSA additives (blue), rac-CSA (red) additive mixtures and in the absence of additives (black) to the Nafion binder under different pH conditions. Each data point represents the mean of three independently prepared electrodes and the error bars represent their standard deviations.

Benchmark OER catalysts, IrO_2_ and RuO_2_, under basic conditions (pH 13.6) were chosen as the catalysts of interest. Figure 1a,b show linear sweep voltammetry (LSV) data in the potential range where oxygen evolution occurs, for IrO_2_ and RuO_2_ nanoparticle catalyst films prepared in a Nafion binder with an S-camphor sulfonic acid (S-CSA, blue) additive, with a racemic mixture of camphor sulfonic acid (rac-CSA, red) additive, and without any additives (black). The data in Figure 1 show a decrease in reaction overpotential for the S-CSA additives compared to that of the rac-CSA additives and the samples with no additive. Atomic force microscope (AFM) topography measurements of the catalyst films comprising S-CSA, rac-CSA, and without additives are not distinguishable from one another (Figure S1). Because the films used in electrochemical measurements are formed from different inks, the LSV data are scaled by the electrochemical surface area (ECSA) rather than the geometric surface area to provide more reliable sample-specific information; see the Supporting Information for more details. Note that for inks prepared without additives, the overpotential at 10 mA cm^–2^ normalized to the geometric area are consistent with that found in other works measured under similar experimental reaction conditions.^28,29^

Unlike previous works in which chiral molecules were designed to adsorb on the catalyst and imprint a chiral bias, the improvement in OER efficiency reported here appears to arise from chiral additives without adsorption onto the surface. IrO_2_ and RuO_2_ films prepared with chiral additives possess similar electrochemical surface areas (0.41 ± 0.07 cm^2^ and 1.2 ± 0.05 cm^2^) to that of films made without additives (0.38 ± 0.05 cm^2^ and 1.2 ± 0.12 cm^2^), which implies that CSA does not influence the density of catalyst active sites; see Table S1. This inference is further supported by circular dichroism measurements of catalyst ink suspensions of S-CSA in Nafion that do not show any chiroptical signatures emanating from the catalyst; see Figure S3 and corresponding discussion. These findings imply that CSA molecules do not adsorb on the catalyst surface, thus alleviating the detrimental effect of adsorbate molecules blocking active sites.

To determine if improved OER performance persists under different pH conditions, additional experiments were performed with our design motif for RuO_2_ and IrO_2_ in 0.5 M H_2_SO_4_; see Figure S10. Similar to that observed under basic conditions, a decrease in reaction overpotential with S-additives was found compared to rac-CSA additives and samples without additives (Figure S10a,b). Table 1 shows how the specific activity of the RuO_2_ catalyst films change with pH and chiral additives. While the magnitude of the specific activity varies considerably with pH, the improvement that arises from chiral additives persists under the different solution conditions; i.e., S-CSA additives show a higher specific activity than the rac-CSA additive or no additive cases. Note that the reduced specific activity of RuO_2_ (Figure S12) at neutral pH conditions is consistent with that shown previously.^30^

**Table 1:** Specific Activity of RuO_2_/Nafion Composites for Different pH Values and Additive Types

	Specific Activity @ η = 350 mV		
pH	No Additive	Rac-CSA	S-CSA
0.4	0.48 ± 0.06	0.65 ± 0.04	0.88 ± 0.04
8	0.021 ± 0.004	0.035 ± 0.003	0.070 ± 0.001
13.6	0.44 ± 0.03	0.60 ± 0.03	0.70 ± 0.02

To further explore the effect of chiral additives for improving the efficiency of OER catalysts, the experimental approach was extended to a state-of-the-art catalyst, Fe_0.7_Co_2.3_O_4_, and measured under basic (1 M NaOH) conditions. Figure 1c shows linear sweep voltammograms, in the potential range where oxygen evolution occurs, for Fe_0.7_Co_2.3_O_4_ nanoparticle catalysts in films prepared with S-CSA additives (blue), rac-CSA additive mixtures (red), and without any additives (black) in a Nafion binder. Similar to the results for IrO_2_ and RuO_2_, achiral Fe_0.7_Co_2.3_O_4_ nanoparticle catalysts show a lowering of the reaction overpotential for catalyst films prepared with chiral additives, compared to achiral additives and matrices made without any additives. Topography mapping of electrodes prepared with S- and rac-CSA additives again are similar (Figure S1). In addition, electrochemical impedance measurements illustrate that the transport resistance during OER is similar for both the S- and rac-CSA additives (see Figure S4a for Nyquist plots and Supporting Information for additional discussion). The improvement in performance for the chiral system over samples without additives is ∼25 mV at J_ECSA_ = 0.5 mA cm^–2^ and provides similar results to that shown with spin promoted catalytic enhancement observed for inherently chiral catalysts over their achiral analogs.^6^ Note that the improved performance is attributed to the enantiopurity of the additive, and the same outcome is reproduced when R-CSA is used in place of S-CSA (Figure S5). The reported enhancement in catalytic activity is further substantiated by a Tafel analysis (37 mV dec^–1^ for S-CSA and 44 mV dec^–1^ for rac-CSA and without additives). Note the data are summarized in Table S2 for all three catalysts. Moreover, the favorable attributes of the chiral additives are found to persist even after 2 h of electrolysis; see Figure S7 for chronopotentiometry measurements, and corresponding LSVs before and after, of achiral Fe_0.7_Co_2.3_O_4_ catalysts with S- and rac-CSA additives. As with IrO_2_ and RuO_2_, ink solutions of the Fe_0.7_Co_2.3_O_4_ catalyst do not show chiroptical signatures and the LSVs were normalized by the ECSA; see Table S1 and Figure S3. Lastly, control experiments show that the increase in observed current with chiral additives does not originate from oxidation of CSA; see Figure S11 and corresponding discussion on control experiments measuring the electrochemical response of CSA.

The mass activity for the achiral Fe_0.7_Co_2.3_O_4_ catalysts with S-CSA additives reported here is 2123 ± 90 A g^–1^ @ η = 350 mV in 1 M NaOH (pH 13.6). Figure S8 shows the linear sweep voltammogram for these Fe_0.7_Co_2.3_O_4_ catalyst composites with S-CSA additives that were used to find the mass activity. This mass activity is even larger than that of the inherently chiral version of the Fe_0.7_Co_2.3_O_4_ catalyst, which we reported to be 1730 ± 178 A g^–1^ @ η = 350 mV previously.^6^ To the best of our knowledge, the reported mass activity represents the highest value to date and implies that the chiral bias emanating from the additives that enhances the OER is on par with, or greater than, that displayed by inherently chiral catalysts.

To further explore the enhancement in the OER of systems with chiral additives, Figure S9 summarizes the specific activity, i.e., the electrochemical surface area normalized current at an overpotential of 350 mV, highlighting an improvement of nearly two times with inks prepared using chiral additives as compared to those with no additive. A similar improvement is observed for RuO_2_ and IrO_2_ in 0.5 M H_2_SO_4_ (Figure S10c,d). Note that the three different catalysts require different preparations for optimal performance, i.e., concentration of the binder and catalyst, solvent composition of the ink, etc.; however, the preparation for a given catalyst is the same with and without additives. Our intent is to illustrate that a chiral bias enhances a given catalyst’s OER performance and not necessarily to compare metrics for different catalysts.

Additional characterization on the effect of chiral additives was performed using rotating ring disk electrode measurements to determine the Faradaic efficiency (see Supporting Information). Figure 1d–f plots the Faradaic efficiency, or fraction of total current associated with O_2_ production, of IrO_2_, RuO_2_, and Fe_0.7_Co_2.3_O_4_ catalyst films prepared with S-CSA additives (blue), rac-CSA additive mixtures (red), and without additives (black) as a function of pH. At high pH, the Faradaic efficiency for chiral, racemic, and no additives is approximately the same; i.e., >90%. As the pH decreases, the catalyst films with chiral additives maintain their high performance (>80%); however, the racemic and no additive analogs display a marked decrease in the Faradaic efficiency. Figure S13 reports a similar phenomenon for analogous measurements made on RuO_2_ under acidic conditions. Such behavior is similar to that reported previously in CISS based OER measurements with chiral electrocatalysts,^6,7,19,20^ and has been attributed to spin-polarized catalyst surfaces inhibiting singlet mediated byproduct formation during electrolysis (e.g., hydrogen peroxide), which becomes more favorable as the pH is reduced. Collectively, these studies imply that chiral additives can improve the performance for a range of OER catalysts and over a range of solution pH conditions.

**Figure 2:**
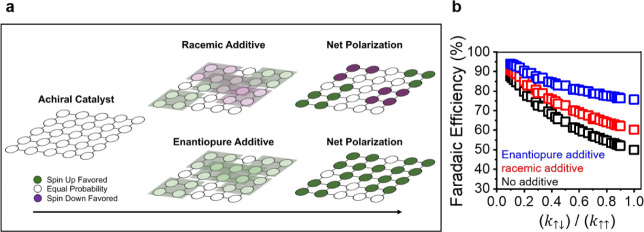
Monte Carlo model on the effect of chiral additives on OER performance. (a) Representative schematic of a catalyst surface in which an increased probability of a given site (indicated by circles) to be spin up or down is represented by green and purple, respectively, and an equal probability to be either spin is shown in white. (Note that in the actual model, 20 × 20 grids of lattice sites, and 3 × 3 site additive domains are used.) The left image shows the case of an achiral catalyst and the middle images show an achiral catalyst in the presence of chiral additives, represented by shaded squares, that are either racemic (top) or enantiopure (bottom). The images on the right represent the net bias on each site resulting from the chiral additives. (b) Plot of the Faradaic efficiency predicted by the model for an achiral catalyst with enantiopure (blue) and racemic additives (red) and without additives (black). Each point is determined from the outcome of 1000 simulations corresponding to a unique combination of model parameters, as discussed in more detail in the Supporting Information.

To understand the mechanism by which chiral additives affect OER efficiency, we developed a simplified Monte Carlo model that describes how the spin distribution of radical intermediates adsorbed on the catalyst surface affects triplet oxygen formation. In this model, adsorption sites on a catalyst are modeled as a two-dimensional square lattice (see Figure 2a), each containing a reaction intermediate whose spin is randomly oriented; either up (green) or down (purple). For an achiral catalyst, in which no external biases influence the spin orientation, each catalyst site has an equal probability for either spin orientation (Figure 2a, left). The addition of chiral molecules in the vicinity of the catalyst are assumed to act as a chiral potential field which biases the spin of the radical intermediates locally in a region of the lattice - i.e., increasing the probability that adjacent lattice sites will have the same spin direction. The domain of influence of a given chiral additive is treated as a square region that overlays several catalyst sites (shaded region, Figure 2a middle) of the underlying catalyst and may overlap with neighboring additives. Both the reaction intermediates (lattice site populations) and the chiral additives were placed randomly on the square lattice, with occupation probabilities determined by the average density of each species. The middle diagram in Figure 2a shows a case for a racemic mixture of additives (top), in which opposite enantiomers exhibit an opposite spin bias, and a case for enantiopure chiral additives (bottom). Note that, for sites with overlapping chiral biases (i.e., with two or more chiral molecules in close proximity) the net bias is treated as an average of the probabilities of each additive to spin polarize a site and ultimately determines the probability of a reaction intermediate at that site for being spin up or spin down (Figure 2a, right) under their joint influence.

In addition to its dependence on the spin populations of two nearest neighbors, the probability of forming a triplet product (via two adjacent reaction intermediate sites with spins aligned parallel) or a singlet byproduct (via two adjacent sites with spins aligned antiparallel) depends on the relative rate of triplet reaction versus the singlet reaction, which can change with catalyst identity and control variables, such as the overpotential. Thus, the reaction yields were calculated as a function of the relative rates for forming singlet () and triplet () reaction products and are reported as a Faradaic efficiency (triplet product/(triplet product + singlet product)). Note that this rate ratio is affected by the bias potential applied during electrolysis such that at high overpotentials both reaction pathways are equally favorable; / = 1. By averaging over many randomly generated populations of spins on the catalyst surface (i.e., one thousand 20 × 20 grids), we calculate a distribution for the fraction of triplet and singlet pairs as a function of the location, enantiopurity, and concentration of molecular additives, coverage of catalyst sites by reaction intermediates, the strength of the chiral bias imparted on the catalyst by the additives, and the preference for a pair of sites to proceed through triplet or singlet coupling. Further details on the implementation of this model and its parameters are discussed in the Supporting Information.

By way of example, consider a case in which reaction intermediates occupy 75% of the available catalytic sites and the probability that a 90% spin polarizing 3 × 3 grid chiral bias is centered at a given site is 20% (spin up for S-additives and spin down for R-additives). Figure 2b shows how the Faradaic efficiency changes as the intrinsic favorability of forming singlet () and triplet () reaction products is varied for enantiopure additives (blue), a racemic mixture of chiral additives (red), and no additives (black). Under all of the varied triplet/singlet coupling probabilities the enantiopure additives show improvements in Faradaic efficiency over those of both the racemic additive mixtures and the case without additives, consistent with that reported in Figure 1d–f. This behavior can be understood as a direct consequence of the chiral bias introduced by the additive. When the additive is a racemic mixture, local alignment of the reaction intermediates within an additive enantiomer’s domain of influence reduces the opportunities for singlet products to form. For sites in which the domains of opposite enantiomers overlap, however, their influences may partially or totally cancel, thus limiting the effective spin polarization (as illustrated in the top row of Figure 2a). Conversely, for enantiopure additives the bias of overlapping additives is the same and therefore does not destructively interfere (as illustrated in the bottom row of Figure 2a). As the relative probability of singlet formation increases, the Faradaic efficiency decreases monotonically in all cases. The influence of an enantiopure chiral bias–that is, Faradaic efficiency higher than that of the racemic mixture or no additive ensembles–is more prominent under these conditions, because fewer singlet coupling pathways exist when the majority of the catalyst surface is spin-polarized.

**Figure 3:**
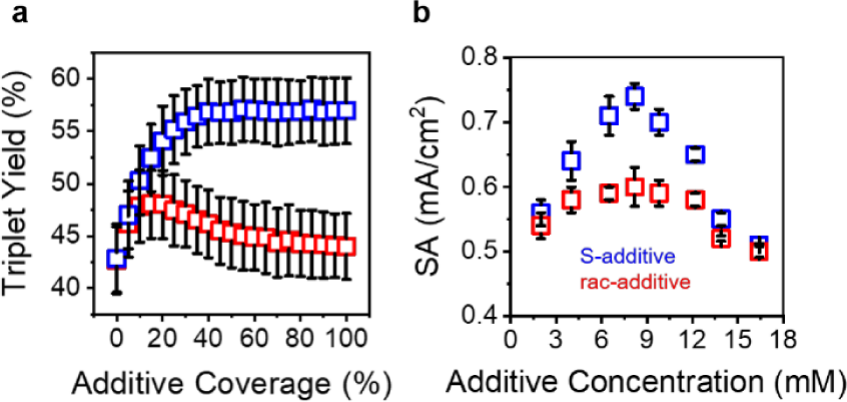
Concentration dependence of additives in OER performance. (a) Monte Carlo simulations of the triplet yield as a function of additive density, demonstrating that enantiopure additives enhance OER better than racemic additives because their influences on reaction intermediates do not conflict with one another. Each point in (a) represents the mean triplet yield calculated from 1000 simulations; error bars represent the associated standard deviations. (b) Experimental measurements of specific activity of Fe_0.7_Co_2.3_O_4_ at 350 mV with S-CSA (blue) and rac-CSA (red), for various degrees of additive loading in the catalyst support. Each data point represents the mean of three independently prepared electrodes and the error bars their standard deviation.

Note that the above model treats the chiral additives as if the molecular orientation in the binder is the same for each molecule; however, in practice the molecules are likely distributed more randomly. Because previous work shows that flipping the dipole moment of a chiral molecule can result in an opposite CISS response,^31,32^ the chiral bias from an enantiopure additive is expected to decrease with orientational averaging, and approach that of a racemic mixture in the absence of any alignment. An important inference that can be drawn from this argument is that some molecular orientation must be present when using CSA as the chiral additive. We also emphasize that, regardless of their orientation, in the limit of low chiral additive concentrations the enantiopure and the racemic mixture should behave the same, since there is minimal overlap between the domains of the additive’s influence, and they are equally capable of aligning reaction intermediate spins locally. Only as additive concentration increases should the data diverge because of the clash in spin preferences between overlapping additives in the racemic case compared with the uniform spin preference between regions when using enantiopure additives. To examine this inference, we used the Monte Carlo model to predict how the triplet yield (here, a proxy for the specific activity since experiments show that triplet oxygen formation is the predominant reaction product at high pH) varies as a function of additive concentration. To reflect the high pH conditions of the experiment, we set the triplet and singlet product formation probabilities to be  = 0.75 and  = 0.25. As before, we assume that the available reaction intermediate sites are populated with 75% probability, and that the “chiral bias” of a single additive is 90%. The model results averaged over multiple simulations are plotted in Figure 3a for additive coverages ranging from 0 to 100%. As expected, at low concentrations the enantiopure additives behave similar to racemic additives. As the concentration of additives is increased, the enantiopure and racemic triplet yields diverge because of the resulting clash of opposing chiral biases.

To corroborate the findings of our model, we investigated the concentration dependence of chiral additives on the specific activity of Fe_0.7_Co_2.3_O_4_ catalysts at high pH (1 M NaOH), i.e., under conditions in which the triplet product formation is favored. Figure 3b shows that at low concentrations (<5 mM) S-CSA additives and rac-CSA additives both increase the specific activity of the catalyst in a similar fashion, but as the concentration of additives is increased (5–9 mM) the S-CSA additives exhibit superior performance to that of rac-CSA. At very large concentrations of additive (>10 mM) the specific activity decreases again and the specific activity for S-CSA and rac-CSA coalesce. We note that the model does not predict the observed decrease in specific activity at higher additive loading, but the behavior is attributed to a decrease in conductivity (see Figure S4b and corresponding discussion) due to excessive organic content present that negates the advantage of enantiopure over racemic additives.

## Conclusions

This work shows that modifying an otherwise achiral electrocatalyst by adding chiral molecules to the conductive support enhances OER efficiency. The behavior is attributed to the additive molecules serving as a chiral bias that spin polarizes the reactive intermediates because of the chiral induced spin selectivity effect. This approach of using small molecule chiral additives offers a flexible strategy through which spin control can be incorporated into existing catalysts without the need of external magnetic fields or the use of chiral ligands that might passivate the catalyst surface. Experiments demonstrated that chiral additives improve the Faradaic efficiency and reduce the reaction overpotential for the oxygen evolution reaction with benchmark catalysts under both acidic and basic conditions. The positive attributes of the chiral additives are preserved even in state-of-the-art electrocatalysts, with studies on these systems leading to remarkably high mass activities. While the experiments in this work are restricted to reactions involving O_2_, many other reactions proceed through radical intermediates that are thought to be or have been proven to be affected by spin control; e.g., oxygen reduction reaction, nitrogen fixation, and carbon dioxide reduction reaction among others.^33−36^ This simple approach for generating spin polarization provides an attractive means for exploring the importance of spin in chemical reactions without the need to synthesize a chiral form for a known catalyst.

## Supporting Information Available

Further experimental details, representative AFM topography of catalyst films, morphology of nanomaterials, representative double layer capacitance measurements, ECSA values for different catalyst films, spectroscopic characterization of chiral imprinting, Nyquist plots, Tafel plots, electrochemical results and analysis, molecular structures, and Monte Carlo model simulation (PDF)
