# Supplementary material for: Improved Catalyst Performance for the Oxygen Evolution Reaction under a Chiral Bias
Source: ACS Catal. 2024 Nov 11;14(23):17303–9. doi: 10.1021/acscatal.4c04477 (PMC11629287; doi:10.1021/acscatal.4c04477)
Supplement: Supplementary file 1 — cs4c04477_si_001.pdf [file cs4c04477_si_001.pdf]

Supplemental information for:

Improved Catalyst Performance for the Oxygen Evolution Reaction Under a Chiral Bias

Aravind Vadakkayil,[1] Wiley A Dunlap-Shohl,[1] Meera Joy,[1] Brian P. Bloom,[1*] and David H. Waldeck[1*]

[1]Chemistry Department, University of Pittsburgh, Pittsburgh, Pa 15260, USA.

Email: bpb8@pitt.edu, dave@pitt.edu

Contents:

1. Materials and Methods

2. Representative AFM topography of catalyst films

3. Representative double layer capacitance measurements

4. ECSA values for different catalyst films

5. Spectroscopic characterization of chiral imprinting

6. Nyquist plots

7. LSVs of $Fe_{0.7}Co_{2.3}O_4$ with S-CSA additives and R-CSA additives

8. Tafel plots

9. Tafel slope of catalyst films

10. Chronopotentiometric measurements

11. Mass activity

12. Specific activity

13. RuO_2 and IrO_2 in acidic pH

14. Cyclic voltammetry of CSA

15. RuO_2 in pH 8

16. Faradaic efficiency of RuO_2 catalyst in acidic conditions

17. Molecular structures

18. Monte Carlo model

19. References

Materials and Methods

Materials – All commercial materials and solvents were used without purification unless otherwise indicated. Sodium borohydride, DL-cysteine, sodium citrate, cobalt (II) chloride, iron (III) chloride hexahydrate, S-camphor sulfonic acid, R-camphor sulfonic acid, Nafion perfluorinated resin solution, IrO_2, and RuO_2 were purchased from Sigma-Aldrich. NaOH and H_2SO_4 were purchased from Fisher Scientific.

Synthesis of $Fe_{0.7}Co_{2.3}O_4$ Catalysts – Iron doped cobalt oxide nanoparticle catalysts were synthesized following previously published protocols.[1,2] Briefly, 15 mL of deionized water (18 MΩ), 2.5 mL of 100 mM $NaBH_4$, 2 mL of 100 mM DL-cysteine, 2 mL of 100 mM sodium citrate, 1 mL of 200 mM cobalt (II) chloride, and 1 mL of 46 mM iron (III) chloride hexahydrate was added to a round-bottom flask and stirred for 2 hours at room temperature. Following stirring, the nanoparticles were precipitated from solution through the addition of isopropanol (7:1 isopropanol to nanoparticle stock solution) and then centrifuged for 20 min at 10,000 rpm. The nanoparticles were then dried and redispersed in water.

Preparation of Catalyst Films – $Fe_{0.7}Co_{2.3}O_4$ ink solutions were prepared by mixing 0.5 mg of S-camphor sulfonic acid (S-CSA) or a racemic mixture of camphor sulfonic acid (rac-CSA) into 12.5 μL of 5 wt% Nafion perfluorinated resin solution and 250 μL of water/isopropyl alcohol (3:1 v/v). The mixture was sonicated for 15 minutes and then 0.5 mg of the catalyst was added to the solution and sonicated for an additional 30 minutes. Once a homogeneous dispersion was formed a 1 μL aliquot was drop cast onto a mechanically polished 0.07 cm^2 glassy carbon electrode and then dried in an oven at 70°C for 30 min to evaporate the solvents. Ink solutions comprising catalysts without additives followed the same procedure, but the CSA was omitted.

IrO_2 and RuO_2 ink solutions were prepared by adding 5 mg of S- or rac-CSA to 10 μL of 5 wt% Nafion and 1 ml of a water/ethanol solution (1:1 v/v). The mixture was sonicated for 15 minutes and then 5 mg of the catalyst, RuO_2 or IrO_2, was added to the solution. An additional 30 min of sonication was performed and a 2 μL aliquot of the solution was drop cast onto a mechanically polished 0.07 cm^2 glassy carbon electrode. The electrode was then dried in an oven at 70 °C for 30 min to evaporate the solvents.

Electrochemical Measurements – Electrochemical measurements were carried out using either a 618 B potentiostat (CH Instruments) or 750 C bipotentiostat (CH Instruments) equipped with a rotating ring disk electrode apparatus (RRDE-3A - ALS). The reference electrode was a saturated KCl Ag|AgCl (CH Instruments) and the counter electrode was a platinum wire. Linear sweep voltammetry (LSV) experiments for all of the catalyst materials were collected at a scan rate of 10 mV s^{-1}. The electrochemical results reported in this work are all *iR* compensated. For the determination of electrochemical surface area (ECSA) in OER experiments, cyclic voltammograms were taken in the non-Faradaic region (0.45 to 0.5 V vs. Ag|AgCl) at scan rates

from 10 to 70 mV s^{-1}. ECSA was calculated using the formula ECSA = C_{dl}/C_s, where C_{dl} is the double layer capacitance obtained from the slope of the current vs. scan rate plot and C_s is the specific capacitance of the material. A specific capacitance value of 35 and 40 μF cm^{-2} was used to calculate the ECSA of nanomaterials in acidic and alkaline conditions, respectively.[3] Electrochemical impedance spectroscopy (EIS) measurements were performed over a frequency range of 0.01–100,000 Hz at a bias potential of 1.56 V vs RHE with an amplitude of 5 mV.

To determine the Faradaic efficiency in OER reactions a rotating ring disk electrode measurement system was employed. Here, a gold ring was used as the analyzer and, to ensure a clean surface, the ring was cycled between -0.03V and 1.41V vs RHE at a scan rate of 500 mV s^{-1} for 50 cycles. Linear sweep voltammetry measurements were then performed at the disk with a 10 mV s^{-1} scan rate at a fixed rotation of 1600 rpm. The ring was held at a constant potential of 0.1 V vs RHE so that the oxygen produced at the disk was reduced at the ring. Measurements were performed in 1 M NaOH, in a 0.1 M sodium carbonate/bicarbonate buffer solution (pH 10), and in 0.02 M phosphate buffer (pH 8). Note, additional measurements for RuO_2 were performed under acidic conditions; 0.5 M H_2SO_4, and in a pH 3 HCl solution. The improvement in Faradaic efficiency associated with additives was calculated using the equation

$$FE = \frac{2 * I_{ring}}{N * I_{disk}}$$

where FE is the Faradaic efficiency, and I_{ring} and I_{disk} are the current densities of the ring and disk electrodes, respectively. N is the collection efficiency for the RRDE and for this ring-disk electrode setup was determined to be 0.37 by control experiments using potassium ferricyanide. The Faradaic efficiency was calculated at a 2 mA cm^{-2} current density (as defined by the geometric area). Each measurement was repeated for at least three independently prepared catalyst electrodes.

Mass activity and specific activity were determined using the following equations: Mass activity = J_{geo}/m and Specific activity = J_{geo}/RF, where m, J_{geo}, and RF refer to mass loading, the geometric current density at 350 mV overpotential, and roughness factor, respectively and RF = ECSA/geometric area of the electrode.

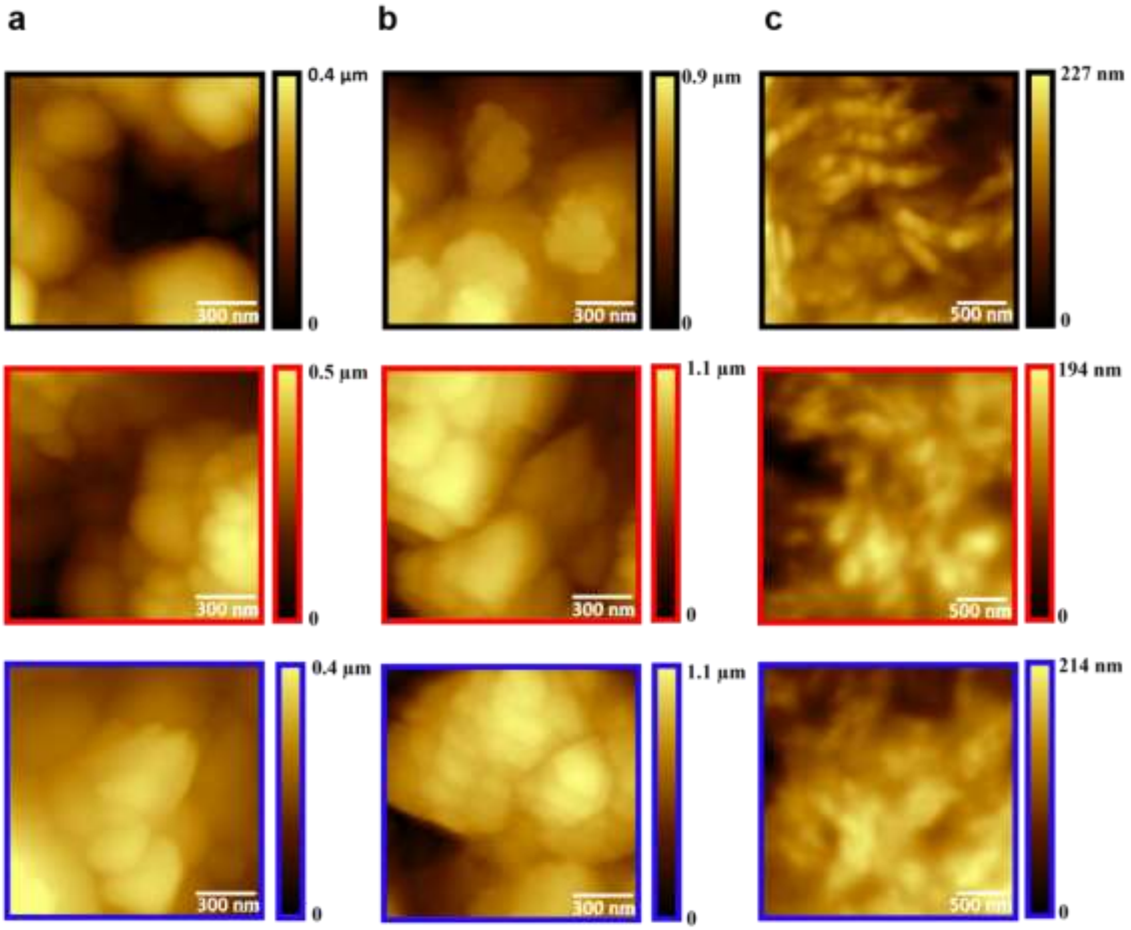

Figure S1. Representative AFM topography of catalyst films. AFM images of IrO_2 (**a**), RuO_2 (**b**), and $Fe_{0.7}Co_{2.3}O_4$ (**c**) catalysts prepared with S-CSA (blue) and rac-CSA (red) additives and in the absence of additives to the Nafion binder (black).

Figure S2 shows representative double layer capacitance measurements from 10 to 70 mV s^{-1} of $Fe_{0.7}Co_{2.3}O_4$ catalysts with S-CSA additives (blue), rac-CSA additives (red), and without additives (black) for determining the electrochemical surface area. The open and filled symbols represent the double layer capacitance when the potential is swept cathodically and anodically, respectively. Table S1 summarizes the experimentally determined ECSA of the different catalyst films investigated.

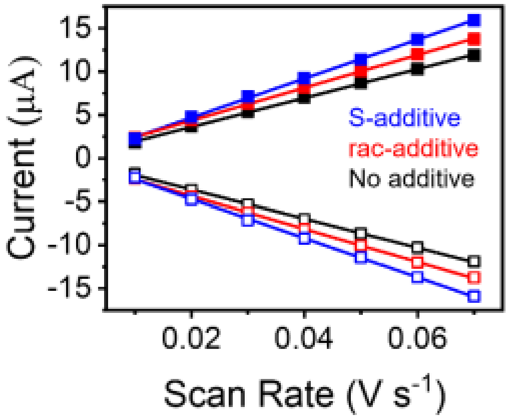

Figure S2. Representative double layer capacitance measurements of $Fe_{0.7}Co_{2.3}O_4$ catalysts with S-CSA additives (blue), rac-CSA additives (red), and without additives (black). The solid line is a linear fit to the data.

Table S1. ECSA values for different catalyst films in different binder-additive combinations. The data represents ECSA averages and error bars of three independently prepared electrodes.

	**S-CSA**	**rac-CSA**	**No additive**
**RuO_2**	1.2 ± 0.05 cm^2	1.1 ± 0.1 cm^2	1.2 ± 0.12 cm^2
**IrO_2**	0.41 ± 0.07 cm^2	0.41 ± 0.01 cm^2	0.38 ± 0.05 cm^2
**$Fe_{0.7}Co_{2.3}O_4$**	5.4 ± 0.12 cm^2	5 ± 1 cm^2	3.5 ± 0.5 cm^2

In order to verify that the improved activity in OER with chiral additives is not associated with imprinting chirality onto the catalysts, spectroscopic measurements on catalyst ink solutions were performed; see Fig. S3. Fig. S3**a** and S3**b** show absorbance and circular dichroism of solutions comprising S-CSA (red), S-CSA and RuO_2 in Nafion (green, dashed line), and S-CSA and IrO_2 in Nafion (blue, dotted line), respectively. Fig. S3**c** and S3**d** show absorbance and circular dichroism of solutions comprising achiral $Fe_{0.7}Co_{2.3}O_4$ catalyst ink suspension in Nafion (black), S-CSA (red), and S-CSA in an achiral $Fe_{0.7}Co_{2.3}O_4$ catalyst ink suspension in Nafion (blue). A sharp transition in all the catalyst solutions prepared with S-CSA occurs to the blue of 325 nm, consistent with control experiments of just S-CSA. These data imply that the CSA is not directly imprinting chirality onto the electronic states of the catalyst; i.e., no new features emerge to the red of 325 nm, where the electronic transitions of the catalysts occur. Please note that in our previous work, where L-cysteine passivated $Fe_{0.7}Co_{2.3}O_4$ catalysts were made, the cysteine imprints chirality onto the catalyst causing Cotton effects to become manifest at the Co(II) → Co(III) intra-particle and surface state (including ligand) → Co(III) optical transitions of the $Fe_{0.7}Co_{2.3}O_4$.[1] The distinct

differences between achiral $Fe_{0.7}Co_{2.3}O_4$ with S-CSA and L-cysteine passivated $Fe_{0.7}Co_{2.3}O_4$ from our previous work clearly demonstrate that the addition of chiral additives does not imprint chirality onto the catalyst's electronic properties.

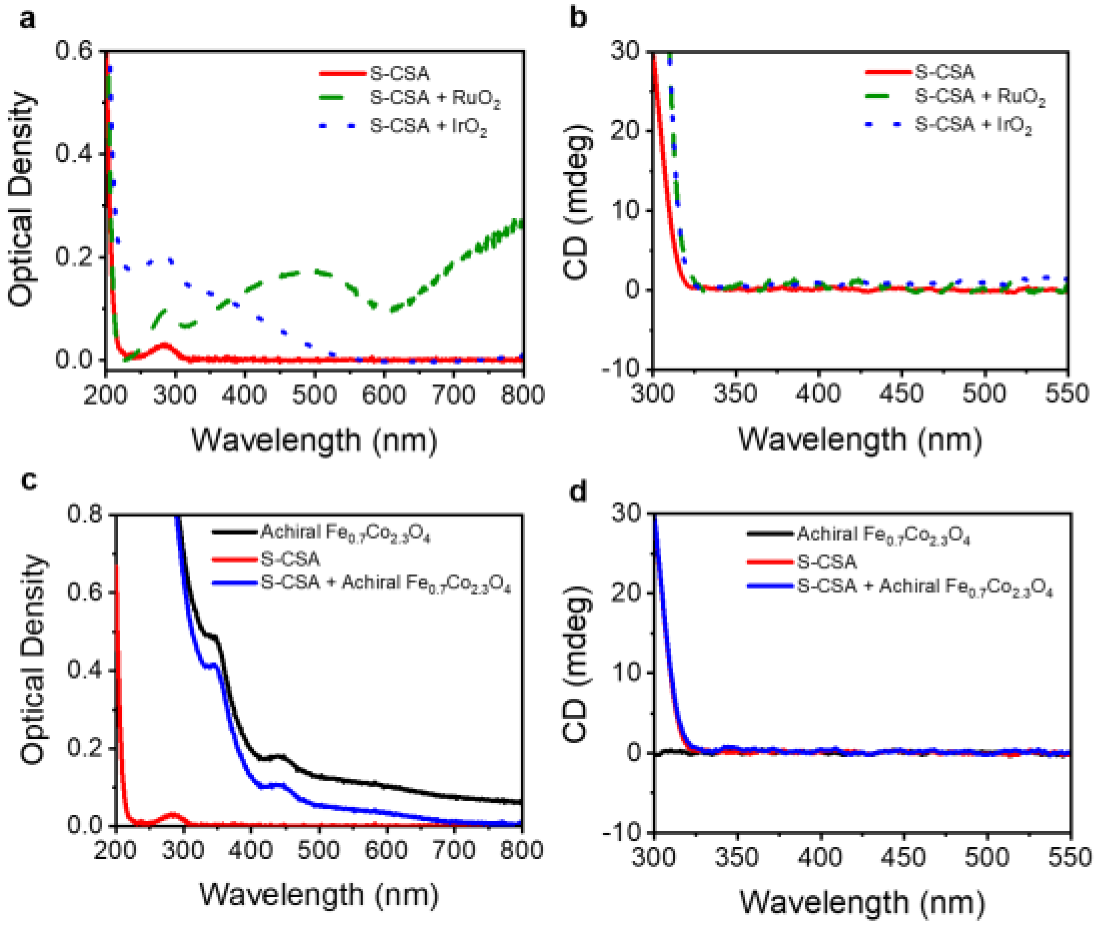

Figure S3. Spectroscopic characterization of chiral imprinting. Absorbance (**a, c**) and circular dichroism (**b, d**) spectra of catalyst suspensions. Panels **a** and **b** show data for S-CSA (red), RuO_2 ink suspensions in Nafion with CSA (green, dashed line), and IrO_2 ink suspensions in Nafion with S-CSA (blue, dotted line). Panels **c** and **d** show data for achiral $Fe_{0.7}Co_{2.3}O_4$ catalyst ink suspension in Nafion (black), S-CSA (red), and S-CSA in an achiral $Fe_{0.7}Co_{2.3}O_4$ catalyst ink suspension in Nafion (blue).

Electrochemical impedance spectroscopy (EIS) data were recorded for $Fe_{0.7}Co_{2.3}O_4$ catalyst with and without additives, within a frequency range of 0.01–100,000 Hz at a bias potential of 1.56 V vs RHE with an amplitude of 5 mV. Fig S4**a** shows that the S-CSA (blue) and rac-CSA (red) additives have similar transport resistance during OER, but they both show decreased transport resistance when compared to the no additive matrix (black). The inset shows the equivalent electric circuit where R_s is the uncompensated solution resistance, Q_{dl} is double-layer charging at the electrode/electrolyte interface in the high frequency domain, R_{ct} is the charge transfer resistance at the electrode/ electrolyte interface, R_p is the pseudo-resistance or resistance related to one or more surface intermediates during the OER, and Q_p is the pseudo-capacitance that represents the change in charged surface species as OER proceeds.[4,5] Fig S4**b** shows representative conductivity measurements at different concentrations of S-additive for $Fe_{0.7}Co_{2.3}O_4$: 0 mM (black), 2 mM (orange), 8.2 mM (blue), 9.8 mM (green), 12.2 mM (grey), and 16.4mM (purple) S-CSA. The OER transport resistance decreases from 0 mM to 8.2 mM of additive concentration; however, beyond that (from 9.8-16.4 mM) the excess additives decrease the conductivity of the matrix. Panel (**c**) shows the change in R_p (pseudo-resistance) as a function of S-additive concentration. The systematic changes in resistance mimic the behavior observed for the specific activity.

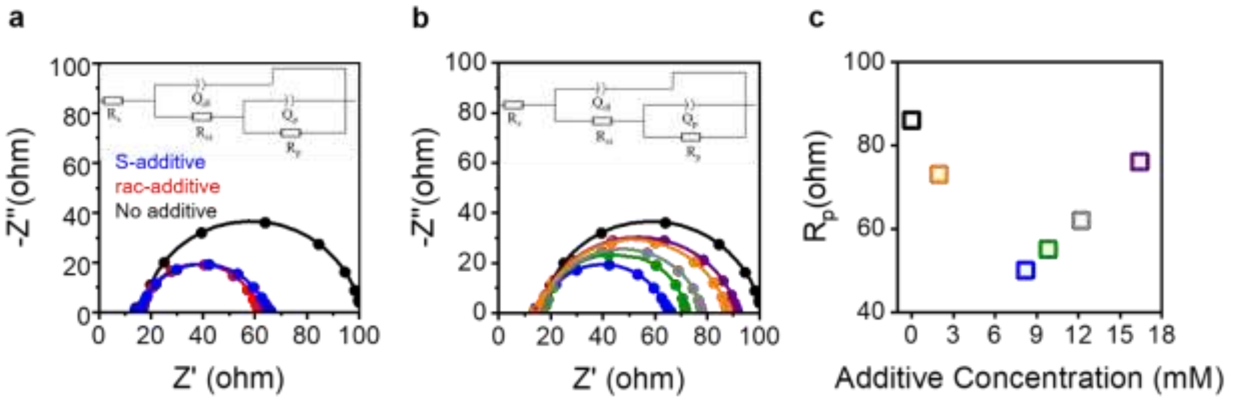

Figure S4. Nyquist plots at a bias potential of 1.56 V vs RHE in the frequency range from 0.01–100000 Hz with 5 mV amplitude for (**a**) $Fe_{0.7}Co_{2.3}O_4$ with S-CSA (blue) and rac-CSA (red) additives and in the absence of additives to the Nafion binder (black). Panel (**b**) shows Nyquist plots for $Fe_{0.7}Co_{2.3}O_4$ at 0 mM (black), 2 mM (orange), 8.2 mM (blue), 9.8 mM (green), 12.2 mM (grey), and 16.4mM (purple) S-CSA additive concentrations. The experimental results are represented by discrete points, and equivalent circuit-fitted results are represented by solid lines. Panel (**c**) is a representative plot of the change in R_p as a function of S-additive concentration. The color coding in Panel (**c**) is the same as that used in panel (**b**). The insets in panels a and b show the equivalent circuits used for the fitting of the data.

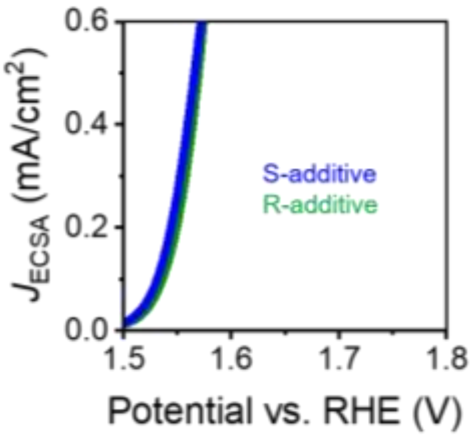

Figure S5. Linear sweep voltammograms of $Fe_{0.7}Co_{2.3}O_4$ catalyst ink suspensions in Nafion with S-CSA additives (blue) and R-CSA additives (green) in 1M NaOH.

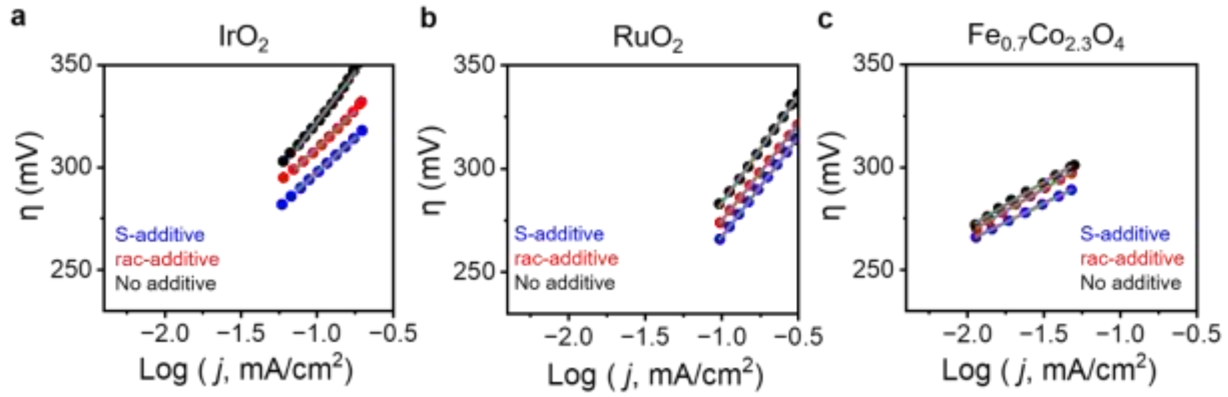

Figure S6. Tafel plots of IrO_2 (**a**), RuO_2 (**b**), and $Fe_{0.7}Co_{2.3}O_4$ (**c**) catalysts prepared with S-CSA (blue) additives, rac-CSA (red) additives, and no additives to the Nafion binder (black). A 1:1 (wt/wt) ratio of catalyst to additive was used for IrO_2 (5mg), RuO_2 (5mg) and $Fe_{0.7}Co_{2.3}O_4$ (0.5mg). Each curve represents the average of three independently prepared electrodes. The solid grey line is a linear fit to the data. The average Tafel slope is reported in Table S2.

Table S2. Tafel slope of catalyst films with and without chiral additives in 1M NaOH**.** The data represent Tafel analysis on the average of three independently prepared electrodes and the error is associated with the quality of the fit to the data.

	**S-CSA**	**rac-CSA**	**No additive**
**RuO_2**	92 ± 2 mV dec^{-1}	92 ± 3 mV dec^{-1}	104 ± 4 mV dec^{-1}
**IrO_2**	67 ± 3 mV dec^{-1}	71 ± 6 mV dec^{-1}	93 ± 5 mV dec^{-1}
**$Fe_{0.7}Co_{2.3}O_4$**	37 ± 4 mV dec^{-1}	43 ± 3 mV dec^{-1}	44 ± 4 mV dec^{-1}

To demonstrate that the added benefit of chiral additives persists for long reaction times chronopotentiometric measurements were performed; electrolysis at a constant current of 10 mA cm^{-2} normalized to geometric area (See Figure S7**a**). Note that the electrolysis was performed under mild stirring conditions to prevent the accumulation of bubbles on the electrode surface. Figure S7**b** and S7**c** show linear sweep voltammograms that were collected before (solid line) and after (dashed line) the electrolysis. The specific activity, at an overpotential of 350 mV, decreases by ~16%, following two hours of electrolysis for both S-CSA (panel **b**) and rac-CSA additives (panel **c**).

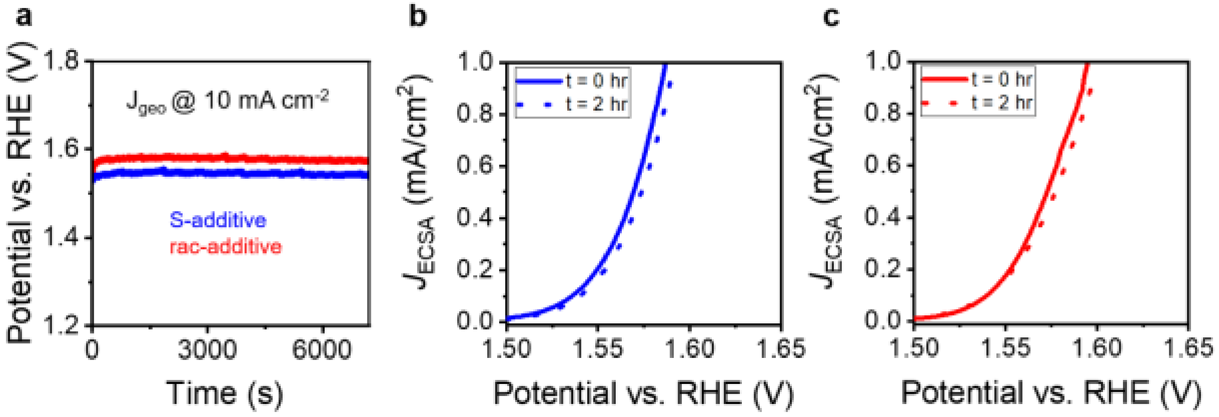

Figure S7. Chronopotentiometric measurements performed at a constant current density of 10 mA cm^{-2} (geometric normalized) for $Fe_{0.7}Co_{2.3}O_4$ catalyst ink suspensions in Nafion with S-CSA additives (blue) and rac-CSA additives (red) for 2 hours (**a**). Linear sweep voltammograms of $Fe_{0.7}Co_{2.3}O_4$ catalyst ink suspensions in Nafion with S-CSA additives (**b**) and rac-CSA additives (**c**) before (solid line) and after (dashed line) 2 hours of electrolysis at a constant current of 10 mA cm^{-2} in 1M NaOH.

Figure S8 shows a geometric area normalized linear sweep voltammogram of achiral $Fe_{0.7}Co_{2.3}O_4$ catalysts with S-CSA additives (blue). The red dashed line indicates an overpotential of 350 mV.

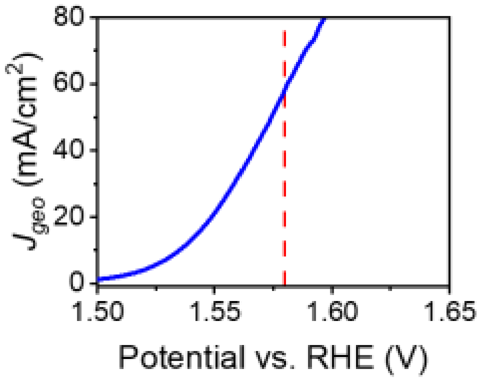

Figure S8. Linear sweep voltammograms of achiral $Fe_{0.7}Co_{2.3}O_4$ catalysts with S-CSA additives normalized to the electrode's geometric surface area. The data represent the average of three independently prepared electrodes and the red dashed line indicates an overpotential of 350 mV.

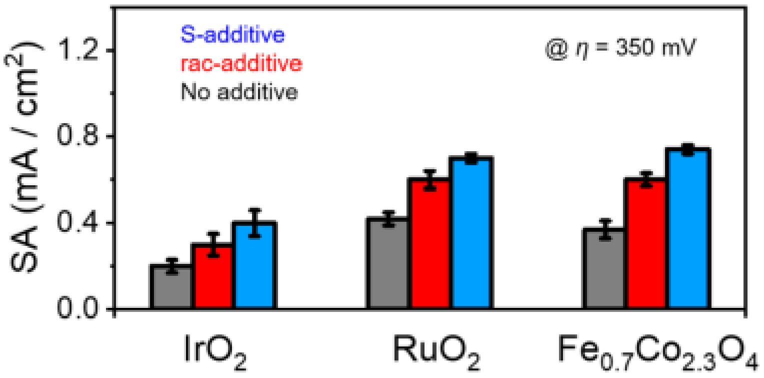

Figure S9. Specific activity (SA) of IrO_2, RuO_2, and $Fe_{0.7}Co_{2.3}O_4$ catalysts prepared with S-CSA (blue) and rac-CSA (red) additives and in the absence of additives to the Nafion binder (black) in 1M NaOH. The error bars in the plot represent the standard deviation to the mean determined from measurements on at least three independently prepared electrodes.

Electrochemical performance of RuO_2 and IrO_2 investigated in acidic conditions with S-CSA (blue) and rac-CSA (red) additives, and without additives (black). The decrease in reaction overpotential and increase in specific activity, are consistent with that reported under basic conditions reported in Figure 1.

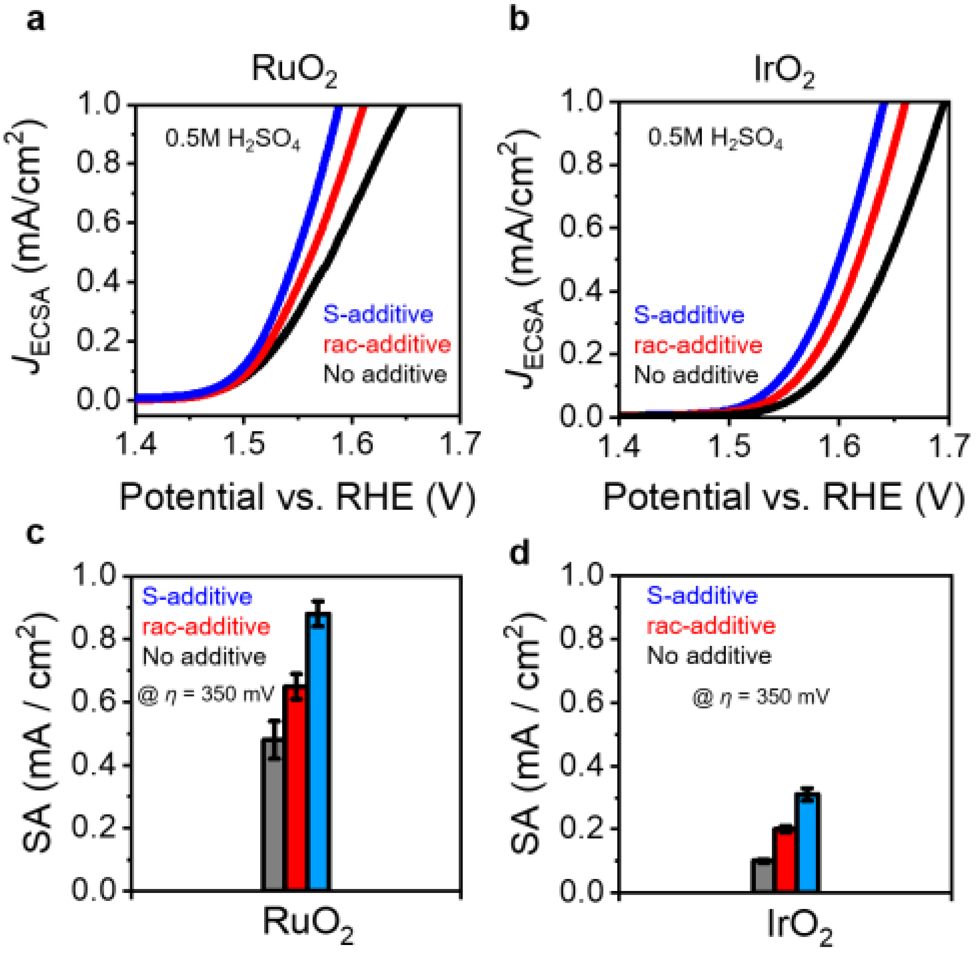

Figure S10. Linear sweep voltammograms of RuO_2 (**a**), and IrO_2 (**b**) catalysts prepared with S-CSA (blue) and rac-CSA (red) additives and in the absence of additives to the Nafion binder (black) measured in 0.5 M H_2SO_4. Each curve in (**a**) and (**b**) represent the average of three independently prepared electrodes. Panels (**c**) and (**d**) plots the Specific activity (SA) of RuO_2, and IrO_2 catalysts prepared with S-CSA (blue) and rac-CSA (red) additives and in the absence of additives to the Nafion binder (black). The error bars in the plot represent the standard deviation to the mean determined from measurements on at least three independently prepared electrodes.

To highlight that CSA does not contribute to the observed current under OER potentials a series of control experiments were conducted. Figure S11 shows cyclic voltammograms of bare glassy carbon electrodes (GCE) under acidic (0.5M H_2SO_4, Panel A) and basic (1M NaOH, Panel B) conditions with (blue) and without (green) 20 mM of CSA in the electrolyte solution. The currents are approximately the same for both systems. To ensure that the catalyst does not facilitate CSA oxidation, additional experiments were also performed on RuO_2 (Panel C) and $Fe_{0.7}Co_{2.3}O_4$ (Panel D) coated electrodes with (blue) and without (green) 20 mM of CSA in the electrolyte solution. No changes to the current response are observed upon addition of CSA and imply that CSA does not contribute to the current response during OER.

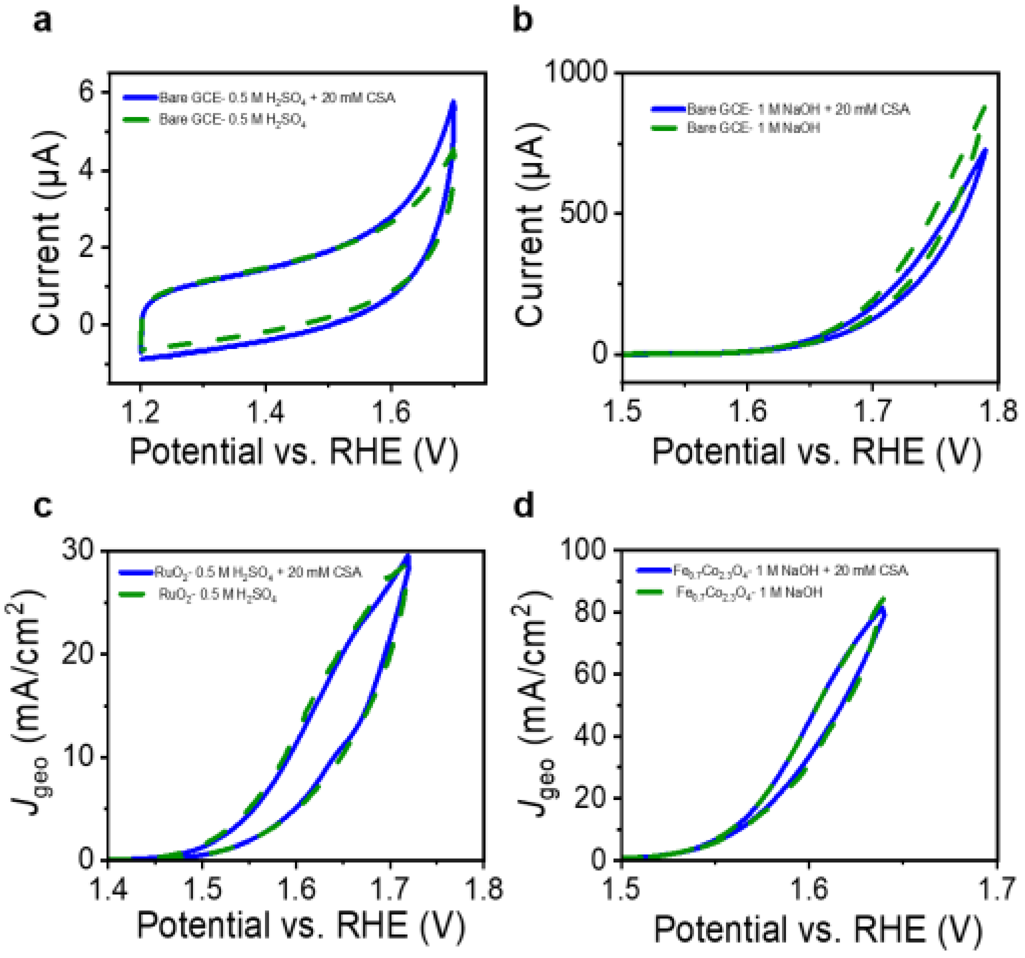

Figure S11. Cyclic voltammograms of a bare glassy carbon electrode in 0.5 M H_2SO_4 (**a**) and in 1M NaOH (**b**) solution with (blue solid line) and without (dashed green line) 20 mM CSA. Panels (**c**) and (**d**) plots representative voltammograms of RuO_2 and $Fe_{0.7}Co_{2.3}O_4$ catalysts with (blue solid line) and without (dashed-green line) 20 mM CSA in 0.5M H_2SO_4 and 1M NaOH, respectively.

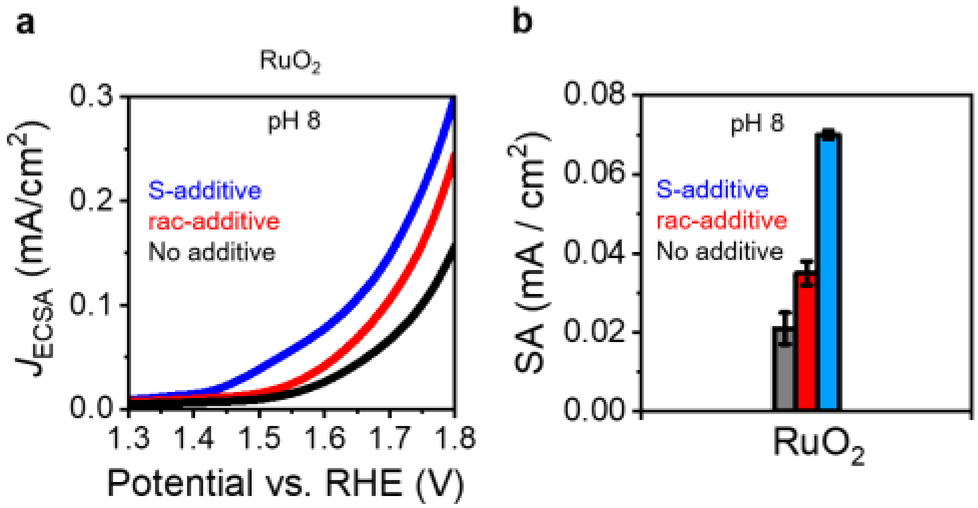

Figure S12. Linear sweep voltammograms (**a**), and Specific Activity (**b**) of RuO_2 catalysts prepared with S-CSA (blue) and rac-CSA (red) additives and in the absence of additives to the Nafion binder (black) measured in 0.02 M phosphate buffer (pH 8). Each data point represents the mean of three independently prepared electrodes and the error bars represent their standard deviations.

Faradaic efficiency of RuO_2 catalyst investigated in acidic conditions

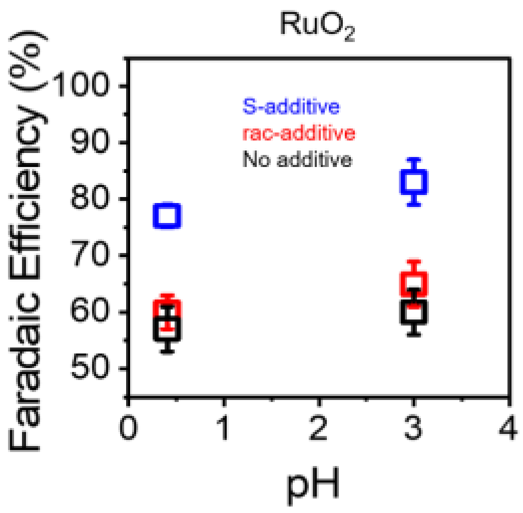

Figure S13. Faradaic efficiency of RuO_2 catalysts prepared with S-CSA (blue) and rac-CSA (red) additives and in the absence of additives (black) to the Nafion binder under acidic pH. Each data point represents the mean of three independently prepared electrodes and the error bars represent their standard deviations.

Table S3. Molecular structures of the binder support (Nafion) and additive (Camphor sulfonic acid).

Binder	Nafion	
	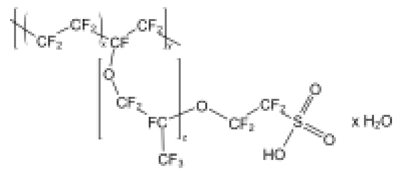	
Additive	S-CSA	R-CSA
	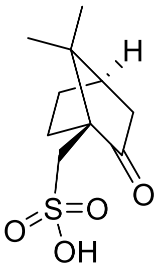	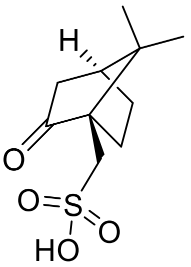

Monte Carlo Model for the influences of chiral additives and catalyst chirality on OER efficiency

The objective of this model is to explain how the Faradaic efficiency of a catalyst is affected by the presence of randomly distributed chiral additives in the vicinity of its surface. To achieve this goal, we begin by considering a patch of catalyst surface as a 20 × 20 square lattice whose nodes represent possible adsorption sites for hydroxyl reaction intermediates, which are occupied randomly with probability θ. Chiral additives may be spatially placed in an overlying grid of the same dimensions according to their average density, and we randomly populate this grid with additives with probability Θ in an analogous manner. The additives' effect on the nearby reaction

intermediates adsorbed at the catalyst surface is determined by each molecule's enantiomeric form, which is also chosen randomly according to the enantiopurity, ε, of the additive ensemble. The combined influence of multiple additives is then used to probabilistically populate the spin states of reaction intermediates on the catalyst surface. In a 3 × 3 grid of lattice sites centered on each additive, the probability that a reaction intermediate is spin-up in this region is 50% + B, where B is the "chiral bias" induced by the additive, and is arbitrarily considered to be positive if the additive is S and negative (with equal magnitude) when the additive is R. When multiple additives affect the same reaction intermediate, the net bias is taken to be the average among them. Finally, adjacent reaction intermediates are given the opportunity to form bonds with adjacent reaction intermediates – either singlet products (e.g., hydrogen peroxide) if the spins are aligned antiparallel, or triplet products (e.g., oxygen) if the spins are aligned parallel. The outcome of these "reactions" are likewise regulated probabilistically; coupling between reaction intermediates which form a bond with their nearest neighbor occur with a rate constant $k_{\uparrow\downarrow}$ for spins aligned antiparallel and $k_{\uparrow\uparrow}$ for spins aligned parallel. After all of the reaction intermediates react, the resulting pairings are used to calculate the singlet and triplet yields for a given patch of catalyst surface, as fractions of paired singlet or triplet species relative to the theoretical maximum of such pairs that could possibly form on the patch. The Faradaic efficiency is then calculated as the ratio of the triplet yield to the sum of the singlet and triplet yields. By constructing many such catalyst patches, we construct distributions of these yields to approximate the ensemble behavior and predict the Faradaic efficiency of the catalyst as a function of the above model parameters.

To construct the plot in Figure 2b, we considered the following combinations of parameters. We fix the reaction intermediate site coverage parameter, θ, at 75% to describe a situation in which a majority of available sites are occupied while retaining some vacancies. We also fix the additive-induced chiral bias enhancement parameter, B, at 40%, such that the additives are mostly effective at polarizing the spins within their domains, but still permit some disorganization. We consider cases for which the additive coverage parameter Θ is either 0% or 20%, and the additive is a racemic mixture or the S-enantiomer. Finally, we consider cases in which both $k_{\uparrow\uparrow}$ and $k_{\uparrow\downarrow}$ take on values from 0 to 100% in increments of 10%, with the restriction that $k_{\uparrow\downarrow}$ is always less than $k_{\uparrow\uparrow}$, reflecting the tendency of both singlet and triplet species to be more strongly favored as the reaction overpotential increases, approaching a limiting case in which their formation probabilities are equal for very high overpotentials. For ease of presentation in Figure 2b, we condense the singlet and triplet formation rate parameters into a single parameter representing their ratio, interpretable as a relative rate of forming singlet products to that of forming triplets. In cases where multiple combinations of ($k_{\uparrow\downarrow}$, $k_{\uparrow\uparrow}$) lead to the same value of $k_{\uparrow\downarrow}/k_{\uparrow\uparrow}$, (e.g., (0.2, 0.4) and (0.4, 0.8)) we report the average Faradaic efficiency among all equivalent cases. For the data presented in Figure 3a, we maintain $\theta = 75\%$ and $B = 40\%$, fix $k_{\uparrow\uparrow} = 0.75$ and $k_{\uparrow\downarrow} = 0.25$, allow Θ to vary from 0 to 100% in increments of 5%, and construct the racemic and pure S cases with simulations conducted with $\varepsilon = 50\%$ and 100%, respectively.

REFERENCES:

[1] Vadakkayil, A.; Clever, C.; Kunzler, K. N.; Tan, S.; Bloom, B. P.; Waldeck, D. H. Chiral electrocatalysts eclipse water splitting metrics through spin control. *Nat. Commun.* **2023**, *14*, 1067.

[2] Yeom, J.; Santos, U. S.; Chekini, M.; Cha, M.; de Moura, A. F.; Kotov, N. A. Chiromagnetic nanoparticles and gels. *Science.* **2018***, 359,* 309–314.

[3] McCrory, C. C. L.; Jung, S.; Peters, J. C.; Jaramillo, T. F. Benchmarking Heterogeneous Electrocatalysts for the Oxygen Evolution Reaction. *J. Am. Chem. Soc*. **2013**, *135*, *45*, 16977–16987.

[4] Aggarwal, P.; Mehra, P.; Paul, A. Pore Size-Regulated Vertically Aligned CoFe-LDH on a Carbon Support for the Oxygen Evolution Reaction. *ACS Appl. Nano Mater.* **2024**, 7, 8, 9532–9541.

[5] Liang, Y.; Banjac, K.; Martin, K.; Zigon, N.; Lee, S.; Vanthuyne, N.; Garces-Pineda, F. A.; Galan-Mascaros, J. R.; Hu, X.; Avarvari, N.; Lingenfelder, M. Enhancement of electrocatalytic oxygen evolution by chiral molecular functionalization of hybrid 2D electrodes. *Nat. Commun.* **2022**, *13*, 3356.
